# High Cardiorespiratory Fitness and Vigorous Physical Activity Relate to Select Pain Sensitivity Assessments in Healthy Adults: A Cross‐Sectional Study

**DOI:** 10.1155/prm/3112089

**Published:** 2026-03-01

**Authors:** Omid Khoshavi, Shannon L. Merkle, Charles J. Paul, Laura A. Frey-Law

**Affiliations:** ^1^ Department of Physical Therapy and Rehabilitation Science, Carver College of Medicine, University of Iowa, Iowa City, Iowa, 52242, USA, uiowa.edu; ^2^ U.S. Army Research Institute of Environmental Medicine, 10 General Greene Avenue, Natick, Massachusetts, 01760, USA; ^3^ McFarland Clinic, 1215 Duff Ave., Ames, Iowa, 50010, USA

**Keywords:** accelerometry, fitness, international physical activity questionnaire (IPAQ), musculoskeletal, quantitative sensory testing

## Abstract

**Background:**

Research highlights the potential role of physical activity (PA) in preventing chronic pain and reducing both acute and persistent pain symptoms. While increasing PA is an evidence‐based intervention for chronic pain, the relationship between pain processing and PA remains unclear. This study aimed to investigate associations of both PA and fitness assessments with multiple measures of pain sensitivity, hypothesizing links with both static and dynamic pain sensitivity metrics.

**Methods:**

Sixty‐four healthy adults (30 female) representing both high and low activity levels completed a series of pressure‐based quantitative sensory tests (QST), including static measures (pressure pain thresholds [PPTs]) and dynamic measures (temporal summation [TS] and conditioned pain modulation [CPM]). PA was evaluated using the International Physical Activity Questionnaire (IPAQ, self‐report) and accelerometry (objective). Cardiorespiratory fitness (CRF) was assessed using the YMCA step test. Correlation and regression analyses were used to evaluate these relationships.

**Results:**

Higher PPTs were related to more self‐reported moderate‐to‐vigorous PA, vigorous PA, and total PA; accelerometry vigorous PA; and high CRF (*p* ≤ 0.01). Self‐reported vigorous PA was inversely correlated with TS (*p* = 0.01), while the other PA or CRF metrics were not significantly associated with either TS or CPM (*p* ≥ 0.04).

**Conclusion:**

CRF or vigorous PA metrics were more consistently related to static pressure QST (PPT) than to dynamic QST (TS and CPM). Our findings in a single cohort mirror the inconsistencies noted across cohorts in the literature, suggesting that PA and CRF exhibit distinct relationships with various QST measures.

## 1. Introduction

Increasingly, both basic science [1, 2] and human observations [3–6] suggest that increased physical activity (PA) helps to prevent chronic pain and reduce the risk of pain incidence and symptoms. However, current activity recommendations are based primarily on evidence demonstrating an inverse relationship between volume of PA and overall risk of chronic disease, disability, and mortality [7]. For example, the Physical Activity Guidelines for Americans suggests all individuals target 150 min of moderate or 75 min of vigorous PA per week, plus 2 days of strengthening exercise [8]. PA is not restricted to purposeful exercise, but is more broadly defined as any energy‐expending movement [9]. Although PA (including exercise) is often recommended to prevent and treat acute and chronic pain conditions, it remains uncertain whether increased PA or higher cardiorespiratory fitness (CRF) is linked to decreased pain sensitivity or psychophysical assessment of pain processing.

Pain sensitivity varies widely among individuals and reflects the resultant balance between peripheral and central mechanisms that produce inhibitory and facilitatory modulation of pain [10, 11], assessed by QST in humans [12, 13]. Pressure pain threshold (PPT), a static QST measurement, assesses deep‐tissue pain sensitivity, while dynamic QST measurements such as conditioned pain modulation (CPM) and temporal summation (TS) evaluate central pain inhibition and facilitation, respectively [14–16]. The presence of CPM is defined as a decrease in pain sensitivity following a painful stimulus (“pain inhibits pain”) [16], and TS is the increase in pain with repeated noxious stimuli [14, 15]. The delicate balance between these two endogenous systems likely influences individual variability in pain perception; a disruption in this balance may predispose an individual to acute or chronic pain conditions [17].

Evidence is mixed regarding the relationships between PA and pain sensitivity as measured by QST. In healthy adults, higher levels of PA were associated with decreased thermal TS [18] or pressure pain sensitivity [19], lower pain unpleasantness [20], and increased thermal CPM [18]. Similarly, when comparing athletes to less fit cohorts, individuals with higher fitness levels exhibited greater CPM [21, 22], increased pain tolerance [21], and reduced pressure pain sensitivity [22, 23]. However, conflicting findings also emerge with no significant associations reported between PA or fitness with either CPM [23, 24] or TS [21, 25].

The observed inconsistencies in prior research may in part be due to the varied QST assessments or the use of different PA metrics [26, 27]. Furthermore, most prior research has relied on cutaneous stimuli to assess CPM or TS responses; however, muscle pain is perceived and processed differently than cutaneous pain [28]. Given that musculoskeletal pain is more common than cutaneous pain and is a significant cause of functional impairment and disability, pressure‐based QST may be more relevant to understanding the relationship between PA, CRF, and pain sensitivity. Additionally, CRF may be more germane to pain sensitivity than a week‐long assessment of PA, as fitness levels may better reflect adaptations from long‐term or habitual activity [29]. Thus, the purpose of this preliminary study was to characterize the relationship effect sizes between static and dynamic deep‐tissue QST with both PA and CRF assessments in a single cohort of individuals.

This information will help clarify observed variability reported for pain sensitivity–PA relations and inform the design of future larger follow‐up investigations. We hypothesized that greater PA and CRF levels would be related to lower static and dynamic pressure pain sensitivity in healthy, pain‐free adults from a range of activity levels.

## 2. Methods

### 2.1. Study Design

This cross‐sectional, observational study was designed to explore meaningful correlations between multiple QST assessments and multiple metrics of PA (self‐report and accelerometry) as well as a measure of CRF in healthy adults spanning both sedentary and active lifestyles. It was conducted in accordance with the STROBE observational study guidelines (see Supporting Information I) [30]. Prior to participation, all individuals provided written informed consent, as approved by the local Institutional Review Board. Participants were compensated for their time.

### 2.2. Participants

To examine the relationships between activity levels and pain sensitivity, we targeted the recruitment of both sedentary and active healthy adults. Participants were recruited via mass email invitations distributed to university graduate students, faculty, and staff; posters in the surrounding community and campus; and a university website of postings for research volunteer opportunities. All participants received compensation for their participation. Inclusion and exclusion criteria were as follows: aged 18–45 years; no chronic pain conditions; no medication use except birth control or multivitamins; no major medical or psychiatric conditions; blood pressure below 140/90 mmHg; and no PA restrictions. Due to the multiple primary outcomes, target sample size was determined generally as having sufficient power (80%) to detect moderate or better correlations (i.e., *r* ≥ 0.387, which corresponds to explaining ≥  15% observed variance). This corresponded to a minimum sample size of 50, which is consistent with mean sample sizes from a number of prior studies [18, 21, 22]. To allow for up to 20% missing or incomplete data, we set a target recruitment of 65.

### 2.3. Procedures

**FIGURE 1:**
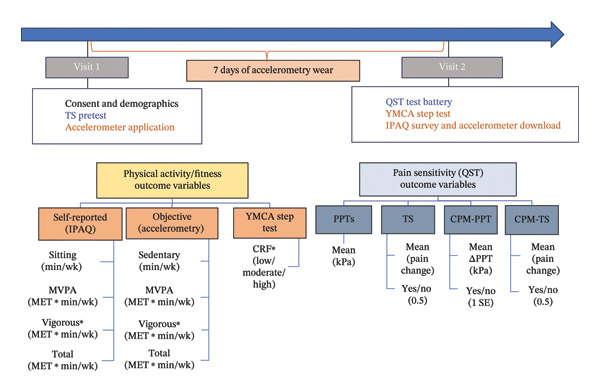
Schematic representation of the study timeline and associated variables extracted for physical activity, cardiorespiratory fitness, and pain sensitivity (QST). ^∗^The primary outcomes. IPAQ, International Physical Activity Questionnaire; CRF, cardiorespiratory fitness; MET, metabolic equivalent; MVPA, moderate plus vigorous physical activity; QST, quantitative sensory testing; PPTs, pressure pain thresholds; TS, temporal summation; CPM‐PPT, conditioned pain modulation with pressure pain threshold; CPM‐TS, conditioned pain modulation with temporal summation.

The study involved two visits separated by 7 days (see Figure 1). At Visit 1, participants completed a demographics survey and the Positive Affect Negative Affect Schedule (PANAS), a valid and reliable measure of positive and negative affect [31]. Participants were then familiarized with the QST procedures in preparation for Visit 2 and fitted with an accelerometer, worn on their nondominant wrist (ActiGraph™ wActisleep + or wActisleep‐BT triaxial, Pensacola, FL). After 7 days, they returned the accelerometers at Visit 2, completed the QST battery, the YMCA step test, and the International Physical Activity Questionnaire (IPAQ)—Long Form.

### 2.4. PA Assessment

Self‐reported PA was assessed using the IPAQ, a widely used, valid, and reliable 7‐day measure of PA across multiple domains: leisure, transportation, work‐related, home, and sedentary [32]. IPAQ data were cleaned and scored according to the 2005 IPAQ guidelines, resulting in activity estimates based on metabolic equivalents (METs) and time. [32]. We extracted four activity metrics (see Figure 1): time spent sitting (min or hr per week), total PA, moderate‐to‐vigorous PA (MVPA), and vigorous PA (MET ∗ min/wk or MET ∗ hr/wk).

Accelerometry PA was assessed continuously for 7 days, including sleep and showering. Participants were instructed to continue their typical activity levels throughout the monitoring period. ActiLife software was used to download the acceleration data and was later processed using custom MATLAB (MathWorks, Inc., R2020b) code to obtain mean accelerations (g) per minute, as previously described [33, 34]. These accelerations (in mg/min) were then transformed to estimates of energy expenditure (in mlO_2_/kg/min) for each minute using a validated, nonlinear form of Hildebrand’s methodology [20, 34] and transformed to METs by a factor of 3.5 [35], given as follows:

(1) VO2=0.901∗wristmg/min0.5343.5.

Four PA metrics were extracted: total activity above sedentary levels: METs > 1.5; MVPA: activity > 3.0 METs; vigorous PA: activity > 6 METs; and time spent sedentary: ≤ 1.5 METs. These metrics were represented as either MET ∗ min/wk, MET ∗ hr/wk, or time (sedentary). For those with < 7 full days of accelerometry data, weekly values were estimated using daily averages multiplied by 7.

Sensitivity analyses were performed considering alternate methods of processing accelerometry using ActiLife processing algorithms (step counts, Freedson’s adult VM3, and Crouter’s VM) with the low frequency extension option, as described previously [36]. Time spent in sedentary, light, moderate, and vigorous were extracted with these alternate methods as well as the above nonlinear algorithm (Equation (1)) for comparison, rather than in MET ∗ min as used in our primary analyses.

### 2.5. CRF Assessment

The YMCA 3‐min, submaximal step test is a practical and reliable assessment used to measure CRF [37]. It does not require major equipment, such as a treadmill or bicycle ergometer, two other common modes of CRF assessment, yet has age‐ and sex‐based normative values for comparison [38]. CRF is determined based on the heart rate recovery immediately following the task, where faster recovery indicates greater fitness. Participants were instructed to step up and down a 12″ step at 24 steps/min for 3 min, using a metronome. Within 5 seconds, participants sat and had their recovery heart rate measured by palpating the radial pulse at the wrist for 1 minute. The cumulative heart rate was scored following YMCA‐reported normative values by age and sex into seven categories: (1) excellent, (2) good, (3) above average, (4) average, (5) below average, (6) poor, or (7) very poor. For the purposes of this study, we then combined these seven to only three categorical CRF groups: high (YMCA Levels 1–2), moderate (YMCA Levels 3–5), and low (YMCA Levels 6–7) to more clearly explore the relationship between large differences in CRF on QST. Because continuous heart rate recovery values are both age‐ and sex‐dependent, we chose to use categorical groupings in our analyses to reduce the potential confounding of results by age and sex [37].

### 2.6. QST

Four psychophysical tests of pain sensitivity were assessed in the following order: PPTs, followed by pressure TS, and lastly two CPM assessments (using PPT and TS as test stimuli) to minimize potential effects of more painful assessments sensitizing less painful assessments. Pain was assessed using a verbal, numeric 0–10 scale (Borg CR10) throughout the QST protocol, where 0 is No Pain and 10 is Maximal Pain. CR10 is a validated scale, which allows individuals to rate pain using any decimal or fractional value, providing more precision when estimating TS than an 11‐pt integer numeric rating scale, similar to 101‐pt numeric pain scales (0–100) while maintaining the more common 0–10 anchors [39, 40].

#### 2.6.1. PPT

PPTs were assessed over the left middle deltoid using a digital, hand‐held pressure algometer (Somedic AB, Sweden) at 30 kPa/s using a 1‐cm^2^ rubber‐tipped probe. Digital algometry is a reliable assessment [41]. Participants were instructed to press the button when the pressure first became painful. PPT trials were separated by the time required to record each value, which was typically under 30 s. While greater time between repetitions minimizes potential sensitization, it is advantageous to complete the PPTs quickly after the cold pressor task to maximize ability to assess for CPM (see Section 2.6.2). The average of three PPT measurements was used as the PPT value.

#### 2.6.2. TS of Pain

TS was assessed using a custom‐built apparatus to apply individualized pressure stimuli with a 1‐cm^2^ tip to the left proximal, dorsal forearm. The pressure was identified at Visit 1, as that which produced moderate pain (3–4/10, CR10) after three stimuli. At Visit 2, participants rated pain after three stimuli at 0.5 Hz using this pressure (initial). Then, 15 pressure stimuli were applied at 0.5 Hz for 30 s. Participants reported their pain intensity at 15 and 30 s during the test and again 30 s afterward. TS was computed as the difference between the maximum pain during or after the test minus the initial pain, where positive values reliably indicate pain facilitation [42]. TS was treated as both a continuous and dichotomous variable, where the presence of TS was defined as a pain increase of ≥ 0.5, thus exceeding the reported minimum detectable change [42].

#### 2.6.3. CPM

Two measures of CPM were assessed, both using cold‐water immersion of the right hand (0°C–2°C circulating water for 60 s) as the conditioning stimulus. One reassessed PPTs (CPM‐PPT) and one reassessed TS (CPM‐TS) following the cold‐water immersion. The order of tests was block randomized to minimize order effects with a 5–10‐min break. CPM was computed as postconditioning minus preconditioning values, where positive CPM‐PPT indicated inhibition, whereas negative CPM‐TS indicated inhibition. Both CPMs were evaluated as continuous and dichotomous variables, where the presence of CPM‐PPT and CPM‐TS was operationally defined as an increase of ≥ 1 standard error [43] in baseline PPT (individualized) or a decrease ≥ 0.5 in TS, respectively, following the conditioning stimuli.

### 2.7. Statistical Analyses

Statistical analyses were conducted using SPSS 22.0 (IBM Corp., Armonk, NY). Summary statistics were computed for all variables, and distribution assumptions were tested. Sex differences were assessed using Mann–Whitney *U* tests or chi‐square tests as appropriate.

To evaluate bivariate relationships between pain sensitivity and self‐report or accelerometry PA metrics, Spearman’s correlations were performed as continuous PA metrics typically exhibit non‐normal distributions. To assess pain sensitivity across CRF levels, an ordered categorical metric, independent‐samples Jonckheere–Terpstra (JT) test for ordered alternatives was used [44], with follow‐up pairwise comparisons as appropriate.

To explore these relationships while adjusting for potential covariates (i.e., sex, positive affect, and negative affect), both linear (PPT) and logistic (dichotomous TS and CPM variables) regression models were evaluated. Covariates were chosen as those demonstrating significant correlation with at least one outcome or predictor variable. Log transformations were employed for outcome variables that did not meet normal distribution assumptions for linear models.

Due to the multiple planned assessments, we identified one metric from each PA/CRF domain as primary: vigorous PA for self‐report and accelerometry and the three‐level category for CRF. We assigned a significance level of *p* ≤ 0.05 to evaluate these primary outcomes, and a more restrictive significance level of *p* ≤ 0.01 for all remaining secondary outcomes: total PA, MVPA, time sitting, or sedentary. This approach reduces Type I error risk without excessively inflating Type II error risk and aligns with prior studies [45]. Lastly, to characterize the inter‐relationships within the PA and CRF metrics, Spearman’s correlation coefficients and JT tests were performed as appropriate.

Sensitivity analyses were performed, repeating the accelerometry analyses with the alternative PA outcomes to determine whether the choice of algorithm substantially influenced the study outcomes.

Microsoft Copilot was used in a limited capacity to edit sentences for clarity and conciseness (word count). All content was originally developed by the authors, and only minor grammatical and/or editorial changes were adopted.

## 3. Results

A total of 69 (33 females, 36 males) adults enrolled in this study ranging in age from 18 to 45 years. Five participants were excluded from all analyses: two withdrew for personal reasons before Visit 2 and three failed to complete the QST assessments. Thus, 64 (30 females, 34 males) completed all pain sensitivity, self‐report PA, and CRF assessments. The mean (SD) time between Visits 1 and 2 was 7.9 (3.4) days. Of the 64 included participants, five had missing data resulting from malfunctioning accelerometers; thus, only 59 (29 females, 30 males) were included in the accelerometry‐related analyses. Demographic characteristics of the included participants are provided in Table 1, where no sex differences were observed for age, body mass index (BMI), ethnicity, or positive affect. Negative affect was higher in males than in females by a small, but significant difference (*p* = 0.04). There were no sex differences observed for any PA metrics (*p* ≥ 0.09) or CRF levels (*p* = 0.49). However, females had greater pressure pain sensitivity, that is, lower baseline PPTs (*p* < 0.001). No sex differences were observed for TS (*p* = 0.81), CPM‐PPT (*p* = 0.09), or CPM‐TS (*p* = 0.38).

**TABLE 1:** Summary participant characteristics (mean ± SD, or *n*, %) for all and by sex.

Characteristics	Total (*n* = 64)	Female (*n* = 30)	Male (*n* = 34)	*p* value
Age (years)	25.4 ± 5.8	25.5 ± 6.1	25.4 ± 5.6	0.79
BMI (m/kg^2^)	24.3 ± 4.5	24.0 ± 4.8	24.5 ± 4.4	0.58
Ethnicity				
Non‐Hispanic, Caucasian	58 (91%)	27 (90%)	31 (91%)	0.87
Affect				
Positive	36.1 ± 6.7	36.6 ± 7.0	35.7 ± 6.5	0.74
Negative	16.6 ± 4.9	15.4 ± 4.5	17.7 ± 4.9	0.04
QST				
PPT (kPa)	268.1 ± 146.7	217.5 ± 147.2	312.6 ± 132.9	< 0.001^∗∗^
TS (0–10)	1.2 ± 1.2	1.1 ± 0.9	1.3 ± 1.3	0.81
CPM‐PPT (kPa)	26.3 ± 73.4	31.6 ± 51.8	21.7 ± 88.8	0.09
CPM‐TS (0–10)	−0.5 ± 1.2	−0.4 ± 1.1	−0.6 ± 1.3	0.38
IPAQ				
Sitting time (hr/wk)	53.0 ± 21.6	50.2 ± 23.2	56.2 ± 20.0	0.09
MVPA (METs ∗ hr/wk)	45.4 ± 38.9	42.2 ± 39.6	48.2 ± 38.7	0.49
Vigorous (METs ∗ hr/wk)	25.2 ± 29.1	22.3 ± 29.3	27.8 ± 29.1	0.29
Total activity (METs ∗ hr/wk)	67.8 ± 49.9	64.2 ± 53.4	70.9 ± 47.3	0.45
Accelerometry^‡^				
Sedentary time (hr/wk)	98.9 ± 20.3	94.7 ± 20.9	102.9 ± 19.3	0.28
MVPA (METs ∗ hr/wk)	67.6 ± 41.7	66.7 ± 43.9	68.4 ± 40.2	0.55
Vigorous (METs ∗ hr/wk)	15.6 ± 25.6	12.2 ± 18.6	18.9 ± 30.8	0.88
Total activity (METs ∗ hr/wk)	179.2 ± 60.4	186.4 ± 64.8	172.6 ± 56.0	0.99
CRF				0.49
Low	28 (44%)	15 (50%)	13 (38%)	
Moderate	12 (19%)	6 (20%)	6 (18%)	
High	24 (37%)	9 (30%)	15 (44%)	

*Note:* CRF, cardiorespiratory fitness. *p* values for sex differences using either Mann–Whitney U or chi‐square.

Abbreviations: BMI = Body Mass Index; CPM = conditioned pain modulation; IPAQ = International Physical Activity Questionnaire; MVPA = moderate‐to‐vigorous physical activity; PPTs = pressure pain thresholds; TS = temporal summation.

^‡^reduced sample size due to missing data (*n* = 59, 29F, 30M).

^∗∗^ *p* ≤ 0.001.

^∗^ *p* ≤ 0.01.

### 3.1. Pain Sensitivity–PA Relationships

#### 3.1.1. PPT

**FIGURE 2:**
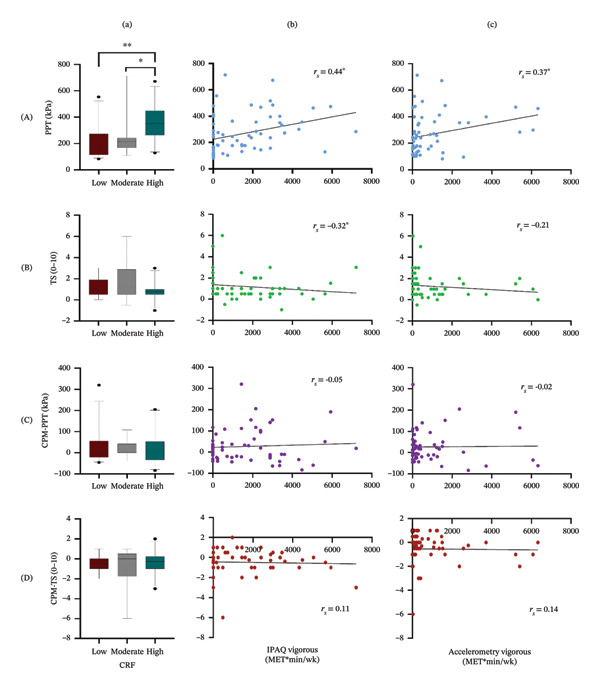
Pain response results by activity and fitness (columns a, b, c) and pain sensitivity measures (rows A, B, C, D) where (a) CRF, cardiorespiratory fitness group (5^th^, 25^th^, 50^th^, 75^th^, and 95^th^ percentiles shown), (b) self‐reported (IPAQ) vigorous physical activity per week, and (c) accelerometry‐measured vigorous physical activity per week; (A) PPT, pressure pain threshold; (b) TS, temporal summation; (C) CPM‐PPT, conditioned pain modulation with pressure pain threshold; and (D) CPM‐TS, conditioned pain modulation with temporal summation. ^∗∗^ *p* ≤ 0.001, ^∗^ *p* ≤ 0.01.

Pressure pain sensitivity demonstrated positive Spearman’s correlations with three of the four self‐reported PA metrics (see Table 2): MVPA (*p* = 0.002), vigorous PA (*p* < 0.001), and total PA (*p* = 0.01), but not sitting time (*p* = 0.65). Whereas using accelerometry, only vigorous PA was associated with PPT (Figure 2, *p* = 0.004), but not sedentary time (*p* = 0.15), MVPA (*p* = 0.41), or total PA (*p* = 0.65).

**TABLE 2:** Spearman’s correlations (*ρ*) between pain measures and activity.

	PPTs	TS	CPM‐PPT	CPM‐TS
IPAQ				
Sitting (min/wk)	−0.06	−0.00	−0.02	−0.06
MVPA (METs ∗ min/wk)	0.39^∗^	−0.20	0.01	0.02
Vigorous (METs ∗ min/wk)	0.44^∗^	−0.32^∗^	−0.05	0.11
Total (METs ∗ min/wk)	0.31^∗^	−0.20	0.03	−0.06
Accelerometry				
Sedentary (min/wk)	0.19	−0.26	−0.24	0.15
MVPA (METs ∗ min/wk)	0.11	0.10	0.07	−0.16
Vigorous (METs ∗ min/wk)	0.37^∗^	−0.21	−0.02	0.14
Total (METs ∗ min/wk)	0.00	0.21	0.15	−0.18

*Note:* MVPA, moderate plus vigorous physical activity.

Abbreviations: CPM‐PPT, conditioned pain modulation with pressure pain threshold, CPM‐TS, conditioned pain modulation with temporal summation; IPAQ, International Physical Activity Questionnaire; PPTs, pressure pain thresholds; TS, temporal summation.

^∗^ *p* ≤ 0.01.

Unadjusted linear regression models indicated that self‐reported MVPA, and self‐reported or accelerometry vigorous PA were each significant predictors of log‐transformed PPT, as the distribution of PPT was not normally distributed (see Supporting Information II, Table S1, *p* ≤ 0.02). However, after adjusting for covariates, only vigorous PA (either self‐reported or accelerometry) remained significant (see Supporting Information II, Table S1, *p* = 0.01 or *p* = 0.05, respectively).

When comparing PPTs across CRF levels, pressure pain sensitivity was lowest (highest PPT) in the high CRF group (Figure 2; JT = 965.00, *p* ≤ 0.002) compared to the low and moderate CRF groups. Both adjusted and unadjusted regression analyses demonstrated that log‐transformed PPTs increased significantly with the increase in the CRF level (see Supporting Information II Table S1, *p* ≤ 0.002).

#### 3.1.2. TS

TS exhibited a low to moderate, negative correlation with self‐reported vigorous PA only (Table 2, *p* = 0.01) but was not significantly related to any other self‐reported (*p* ≥ 0.11) or accelerometry PA variables (*p* ≥ 0.04). Using logistic regression to predict incidence of TS, no PA or CRF metrics were significant with or without adjustment for sex or affect (see Supporting Information II, Table S2, *p* ≥ 0.02). Lastly, TS did not differ significantly between CRF groups (Figure 2; JT = 538.00, *p* = 0.16).

#### 3.1.3. CPM

Spearman’s correlations revealed there were no significant relationships between CPM‐PPT or CPM‐TS and any of the self‐reported PA (Table 2, *p* ≥ 0.72 and *p* ≥ 0.38, respectively) or accelerometry metrics (*p* ≥ 0.06 and *p* ≥ 0.17, respectively). Considering the incidence of CPM using logistic regression, again, no PA metrics were significant predictors with or without adjustment (see Supporting Information II, Table S3, *p* ≥ 0.07 for CPM‐PPT and Table S4, *p* ≥ 0.09 for CPM‐TS). Lastly, CPM did not vary across CRF levels (Figure 2) for either CPM‐PPT (JT = 641.00, *p* = 0.93) or CPM‐TS (JT = 662.50, *p* = 0.85) based on JT analysis or logistic regression (see Supporting Information II, Tables S3 and S4, *p* ≥ 0.39).

### 3.2. Activity and CRF Metric Relationships

Correlations between self‐reported and accelerometry metrics (see Supporting Information II, Figure S1) were significant for MVPA, vigorous PA, and total PA (*p* ≤ 0.01), at low to moderate magnitudes. However, self‐reported sitting was not significantly correlated with accelerometry sedentary time (*p* = 0.29). When comparing self‐reported and accelerometry PA metrics across CRF categories, the high CRF group showed significantly greater vigorous and MVPA (see Supporting Information II, Figures S2 and S3) than the low and moderate CRF groups. Self‐reported (*p* < 0.001), but not accelerometry (*p* = 0.17), total PA was greater in the high CRF than in the low and moderate CRF groups. Similarly, self‐reported (*p* = 0.01), but not accelerometry (*p* = 0.66), sedentary time was less in the high CRF than in the other groups.

### 3.3. Alternate Accelerometry Sensitivity Analysis

When repeating the correlations between QST and accelerometry using the alternate processing algorithms, similar results were observed. Time spent in vigorous activity remained significantly related to PPT (*r* = 0.35, *p* = 0.006). None of the other PA–QST correlations were significant (see Supporting Information II, Table S5).

## 4. Discussion

To our knowledge, this is the first study to comprehensively assess CRF, multiple subjective and objective PA measures, and static and dynamic pain sensitivity in healthy adults. Thus, we were uniquely able to evaluate the consistency of these pain sensitivity relationships with various PA assessments and CRF in a cohort with varying levels of usual PA. Our findings partially support our primary hypotheses that vigorous PA and higher CRF level would be most strongly linked to less pain sensitivity. PPTs were associated with higher CRF and vigorous PA from both self‐reported and accelerometry assessments, whereas dynamic QST showed few associations with activity or CRF level; TS was only associated with vigorous self‐reported PA, and no significant relationships were found with CPM.

A higher proportion of studies found stronger associations between PPT with high fitness [22, 23, 26, 46] or self‐reported vigorous PA [19] than with accelerometry metrics [47, 48], consistent with our findings. Longitudinal studies similarly showed that low‐intensity exercise did not alter PPT [49, 50], while vigorous exercise [51] was linked to reduced pressure pain sensitivity. Yet, others found vigorous PA was not associated with PPTs at the temporalis muscle or finger [25, 47], potentially reflecting differences from the QST assessment site [52]. This may suggest that limb muscles are more influenced by regular PA than either a facial muscle or a bony site such as the finger. The functional diversity of nociceptors across different tissues [53], alongside increased capillary density in PA‐activated skeletal muscles [54], may account for the observed tissue‐specific responses. These peripheral muscle adaptations are likely involved in pain sensitivity, providing plausible explanations for peripheral and not simply central mechanisms.

Several mechanisms have been proposed to explain the impact of PA and fitness on pain sensitivity. Higher levels of PA and fitness enhance resilience and stress tolerance by modulating neural processes underlying cognition [55, 56]. In turn, greater resilience and endurance are linked to lower pain sensitivity, as they help individuals shift attention away from pain toward adaptive coping strategies [57]. Furthermore, PA contributes to reducing allostatic load by lowering metabolic risk factors [58], which is related to the nociceptive flexion reflex [59]. Higher CRF resulting from exercise training is linked to lower levels of visceral and peripheral fat [60, 61]. Adipose tissue releases pro‐inflammatory adipokines, while muscle tissue releases anti‐inflammatory myokines [62]. Pro‐inflammatory mediators contribute to peripheral sensitization by binding to nociceptor receptors and increasing their excitability [63]. The collective impact of these factors on peripheral tissues, including joints and muscles, may contribute to the observed differences in static pain sensitivity with vigorous PA or between CRF levels [64]. That is, the modulation of peripheral pain processing through alterations in cytokine levels and muscular adaptations may contribute to reduced pressure pain sensitivity [65]. However, the peripheral inflammatory system effects may not necessarily alter the central processing of pain. While research suggests that regular PA can produce adaptations in brain function and morphology [66, 67], our findings did not reveal a consistent difference in TS or CPM in individuals with greater PA or fitness levels. This could be because brain function and morphology are not always tightly coupled to CNS processing of pain [68] or that the inherent variability in dynamic QST is substantial enough to mask small effects related to an active lifestyle. While adaptations to the immune system could impact both peripheral and central mechanisms, our results may suggest a more direct influence on localized tissues, such as PPTs, rather than on central pain processing pathways, which may explain the lack of differences observed in TS or CPM with PA or CRF levels.

Although the dynamic QST measures are thought to reflect central pain processing influences and thus potentially have comparable or stronger associations with activity and fitness levels than static QST measures [24], our findings indicted that pressure TS was only correlated with self‐reported vigorous PA, but not other activity level variables, reducing our confidence in this isolated finding. Prior studies have been mixed in this regard, with several reporting weak or no significant relationships between dynamic QST and PA or fitness metrics [23, 25, 27, 69], whereas others have found significance for one or more dynamic QST measures [18, 21, 24, 47, 69].

Methodological variations might help explain these conflicting findings. In addition to the specificity of PA–pain sensitivity relationships depending on anatomical structure discussed above, differences may also arise from the nature of the noxious stimulus. Literature suggests that PPTs are more closely linked with PA and/or fitness [19, 22, 23, 26, 46] compared to thermal pain thresholds [18, 21, 24, 46]. However, the opposite seems true for dynamic QST. Several studies employing thermal or electrical stimuli reported lower TS [18, 24, 69] and/or greater CPM [18, 21, 47, 69] in highly active healthy individuals. However, those employing pressure stimuli often failed to detect significant relationships with dynamic QST [23, 25, 27, 69]. Indeed, cutaneous and deep‐tissue noxious stimuli activate distinct regions in the dorsal horn [70] and thus may activate different interneurons along their pathways within the central nervous system. Collectively, these findings suggest that central pain mechanisms are not independent from the initial site of activation in the spinal cord, potentially influencing central pain processing and modulation of pain signals [71]. Our findings, along with this body of research, might suggest that QST assessments should be interpreted with caution when used as single indicators of central pain processing. While each may serve as a marker of central pain processing, the assumption that they provide equivalent information despite the range of neural pathways involved in pain processing is likely an oversimplification. Thus, each QST may provide unique insights into pain sensitization vulnerabilities or underlying mechanisms. Future efforts to refine our understanding of each QST assessment and/or develop more robust QST protocols, that minimize variability while improving the precision of pain sensitivity assessment, may be worthwhile.

We observed reasonable agreement between self‐reported and accelerometer‐measured PA, aligning with previous research [47, 72]. However, multiple self‐reported PA metrics showed significant differentiation between CRF levels, whereas for accelerometry, only MVPA and vigorous PA differed by CRF levels. This may be due to the assessment of incidental PA that occurs with daily living activities that are captured with accelerometry but not self‐report, which focusses on largely purposeful activity. Apparently, purposeful and vigorous forms of PA align most closely with mechanical pain sensitivity, particularly PPTs.

Our findings suggest that self‐reported PA may serve as an acceptable scale for studying pain sensitivity among healthy adults. Similarly, the Tromso study found self‐reported activity was correlated with thermal pain sensitivity, both cross‐sectionally and longitudinally, whereas accelerometry outcomes were not [73]. However, other smaller observational study has concluded the opposite [20]. Accelerometry outcomes are influenced by various factors such as wear site, or choice of analysis algorithm and filters used to process the data [36, 74]. However, our sensitivity analyses considering alternate processing methods of the accelerometry signals did not substantially alter our findings.

Several factors may limit the generalizability of our findings. Although CRF levels were based upon sex‐differentiated, validated norms of heart rate recovery using a constant‐rate, standard‐height, step test [37], it provides only an indirect estimate of fitness as it does not directly measure maximal oxygen uptake. Future studies exploring the relationship between muscle strength (as another fitness indicator) and pain sensitivity could also yield valuable insights. Another limitation of this study is the potential influence of the intertrial interval on PPT measurements. While the literature suggests at least 30 s between trials to avoid TS [75], PPTs were repeated more quickly than this to minimize the total test time. Furthermore, some might argue that the lack of significant findings is due to inadequate power. However, our study detected small effect sizes (e.g., difference in sitting time between CRF levels), indicating adequate power to detect meaningful differences. Additionally, our findings align with larger studies that also failed to show significant associations between various PA or fitness metrics with CPM or TS [23, 25, 27]. Interestingly, self‐reported PA was more strongly correlated with QST measures than accelerometry. Certainly, self‐report is at higher risk for recall bias [76]; however, accelerometry is an indirect method that is not immune to errors due to inability to assess static muscle workloads and effects of adding mass to the arm (e.g., carrying bags or purses). In fact, self‐reported PA metrics were more clearly differentiated by CRF level than were accelerometry metrics. Lastly, recruitment from a healthy adult population may not generalize to older adults, those with health conditions or with chronic pain who may have a different PA or fitness relationship with QST. Evidence suggests that despite impaired CPM [77] and exercise‐induced hypoalgesia [78] in chronic pain patients, optimal PA load may still help to reduce pain sensitivity, compared to the sedentary lifestyle [77]. However, our results may infer that effects of PA or fitness do not necessarily affect all forms of pain sensitivity assessment equally.

## 5. Conclusions

Our findings show that CRF and vigorous PA are related to select measures of pain sensitivity. Stronger associations were observed between more vigorous PA and CRF levels with PPTs compared to TS and CPM. Due to the many methods available to assess PA, fitness, and pain sensitivity, the observed inconsistencies across prior studies using isolated assessments of PA or fitness have made interpretation challenging. We show that there is a degree of dependence on the PA assessment method used, as well as on the pain sensitivity construct considered. While there was little association between activity level or CRF with dynamic pain sensitivity, increased PA and/or fitness was never linked to increased static or dynamic pain sensitivity in pain‐free adults. We conclude that greater PA, that is, PA sufficient to produce increased fitness levels, does not affect all forms of pain sensitivity equally, but rather shows the greatest relation to reduced pressure pain sensitivity.

## Funding

No funding was received for this manuscript. Support for C.J.P. was provided by the Iowa Medical Student Research Program (IMSRP).

## Disclosure

A preliminary form of this study was presented in abstract form at the U.S. Association for the Study of Pain in 2025 [79].

## Conflicts of Interest

The authors declare no conflicts of interest.

## Supporting Information

STROBE Checklist for observational studies and additional figures and tables of results are provided in the Supporting Information.

## Supporting information


**Supporting Information** Additional supporting information can be found online in the Supporting Information section.

## Data Availability

The physical activity data are available as part of a larger dataset of objective and self‐report activity data made publicly available [80]. The pain sensitivity data collected for this cohort are available upon request from the corresponding author.
