# Supplementary material for: High Cardiorespiratory Fitness and Vigorous Physical Activity Relate to Select Pain Sensitivity Assessments in Healthy Adults: A Cross‐Sectional Study
Source: Pain Res Manag. 2026 Mar 1;2026:3112089. doi: 10.1155/prm/3112089 (PMC12950830; doi:10.1155/prm/3112089)
Supplement: Supplementary file 1 — Supporting Information Additional supporting information can be found online in the Supporting Information section. [file PRM-2026-3112089-s001.zip › Supplemental Materials I.pdf]

High cardiorespiratory fitness and vigorous physical activity relate to select pain sensitivity assessments in healthy adults: A cross-sectional study

Supplemental Materials I

STROBE Statement—checklist of items that should be included in reports of observational studies

	Item No.	Recommendation	Page No.	Relevant text from manuscript
**Title and abstract**	1	(*a*) Indicate the study's design with a commonly used term in the title or the abstract	1-2	**In both title & abstract**
		(*b*) Provide in the abstract an informative and balanced summary of what was done and what was found		
**Introduction**				
Background/rationale	2	Explain the scientific background and rationale for the investigation being reported	3-4	**Introduction**
Objectives	3	State specific objectives, including any prespecified hypotheses	4	**Introduction**
**Methods**				
Study design	4	Present key elements of study design early in the paper	4-5	**Study design section**
Setting	5	Describe the setting, locations, and relevant dates, including periods of recruitment, exposure, follow-up, and data collection	6	**Procedure**
Participants	6	(*a*) *Cohort study*—Give the eligibility criteria, and the sources and methods of selection of participants. Describe methods of follow-up<br>*Case-control study*—Give the eligibility criteria, and the sources and methods of case ascertainment and control selection. Give the rationale for the choice of cases and controls<br>*Cross-sectional study*—Give the eligibility criteria, and the sources and methods of selection of participants	5	**In Participants section**
		(*b*) *Cohort study*—For matched studies, give matching criteria and number of exposed and unexposed<br>*Case-control study*—For matched studies, give matching criteria and the number of controls per case	n/a	
Variables	7	Clearly define all outcomes, exposures, predictors, potential confounders, and effect modifiers. Give diagnostic criteria, if applicable	6-9	Procedure including ***Physical Activity Assessment, Cardiorespiratory Fitness Assessment, Quantitative***

				Sensory Testing (QST.
Data sources/ measurement	8*	For each variable of interest, give sources of data and details of methods of assessment (measurement). Describe comparability of assessment methods if there is more than one group	6-9	Procedure including ***Physical Activity Assessment, Cardiorespiratory Fitness Assessment, Quantitative Sensory Testing (QST.***
Bias	9	Describe any efforts to address potential sources of bias	6 and 9	**Procedure and Statistical Analysis**
Study size	10	Explain how the study size was arrived at	4-5	**Addressed in Study design**
Quantitative variables	11	Explain how quantitative variables were handled in the analyses. If applicable, describe which groupings were chosen and why	6-10	**Procedure and Statistical Analysis**
Statistical methods	12	(*a*) Describe all statistical methods, including those used to control for confounding	9-10	**Statistical Analysis**
		(*b*) Describe any methods used to examine subgroups and interactions	9-10	**Statistical Analysis**
		(*c*) Explain how missing data were addressed	9-10	**Statistical Analysis**
		(*d*) *Cohort study*—If applicable, explain how loss to follow-up was addressed<br>*Case-control study*—If applicable, explain how matching of cases and controls was addressed<br>*Cross-sectional study*—If applicable, describe analytical methods taking account of sampling strategy	9-10	**Described in Methods (targeting sedentary and active adults) and Statistical Analysis**
		(*e*) Describe any sensitivity analyses	10	**Statistical Analysis (considered 3 approaches for analyzing accelerometry data)**
**Results**				
Participants	13*	(a) Report numbers of individuals at each stage of study—eg numbers potentially eligible, examined for eligibility, confirmed eligible, included in the study, completing follow-up, and analysed	10	**Results**
		(b) Give reasons for non-participation at each stage	10	**Results**

		(c) Consider use of a flow diagram	n/a	**Only 2 visits**
Descriptive data	14*	(a) Give characteristics of study participants (eg demographic, clinical, social) and information on exposures and potential confounders	10	**Results and Table 1**
		(b) Indicate number of participants with missing data for each variable of interest	10	**Results**
		(c) *Cohort study*—Summarise follow-up time (eg, average and total amount)	n/a	
Outcome data	15*	*Cohort study*—Report numbers of outcome events or summary measures over time	*n/a*	
		Case-control study—Report numbers in each exposure category, or summary measures of exposure	*n/a*	
		Cross-sectional study—Report numbers of outcome events or summary measures	10	**Explain outcomes in Methods, report outcomes in Results**
Main results	16	(*a*) Give unadjusted estimates and, if applicable, confounder-adjusted estimates and their precision (eg, 95% confidence interval). Make clear which confounders were adjusted for and why they were included	12-15	**Results and Supplemental Materials II**
		(*b*) Report category boundaries when continuous variables were categorized	12-15	**Predetermined fitness category boundaries in Methods**
		(*c*) If relevant, consider translating estimates of relative risk into absolute risk for a meaningful time period	n/a	
Other analyses	17	Report other analyses done—eg analyses of subgroups and interactions, and sensitivity analyses	n/a	(see above for sensitivity)
**Discussion**				
Key results	18	Summarise key results with reference to study objectives	15-16	**Discussion**
Limitations	19	Discuss limitations of the study, taking into account sources of potential bias or imprecision. Discuss both direction and magnitude of any potential bias	18	**Discussion**
Interpretation	20	Give a cautious overall interpretation of results considering objectives, limitations, multiplicity of analyses, results from similar studies, and other relevant evidence	16-18	**Discussion**
Generalisability	21	Discuss the generalisability (external validity) of the study results	18-19	**Discussion**
**Other information**				

High cardiorespiratory fitness and vigorous physical activity relate to select pain sensitivity assessments in healthy adults: A cross-sectional study

Supplemental Materials I

Funding	22	Give the source of funding and the role of the funders for the present study and, if applicable, for the original study on which the present article is based	20	***Conflict of interest***
