# Supplementary material for: High Cardiorespiratory Fitness and Vigorous Physical Activity Relate to Select Pain Sensitivity Assessments in Healthy Adults: A Cross‐Sectional Study
Source: Pain Res Manag. 2026 Mar 1;2026:3112089. doi: 10.1155/prm/3112089 (PMC12950830; doi:10.1155/prm/3112089)
Supplement: Supplementary file 1 — Supporting Information Additional supporting information can be found online in the Supporting Information section. [file PRM-2026-3112089-s001.zip › Supplemental Materials II.pdf]

High cardiorespiratory fitness and vigorous physical activity relate to select pain sensitivity assessments in healthy adults: A cross-sectional study

Supplemental Materials II

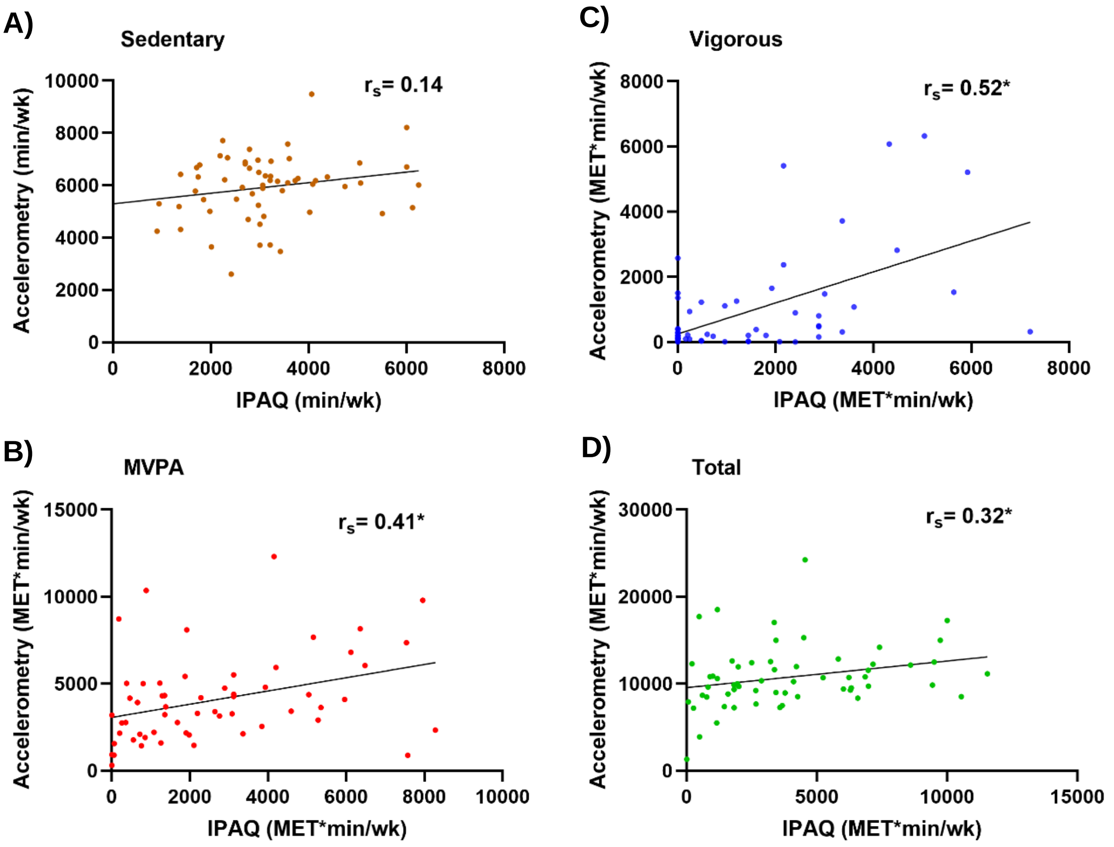

Figure S1. Accelerometry-measured physical activity (PA) versus self-reported activity (IPAQ) in 4 representative activities: A) Sedentary time; B) Moderate plus vigorous PA (MVPA); C); Vigorous PA and D) Total PA. *$p \leq 0.01$.

High cardiorespiratory fitness and vigorous physical activity relate to select pain sensitivity assessments in healthy adults: A cross-sectional study

Supplemental Materials II

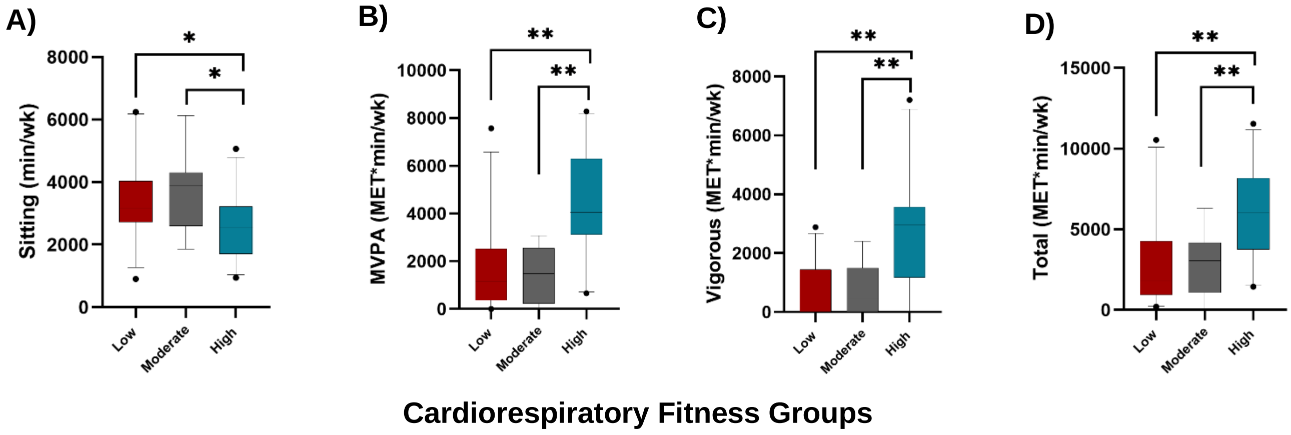

Figure S2. Box plots (5th, 25th, 50th, 75th, and 95th percentiles shown) of self-reported activity (IPAQ) of participants by cardiorespiratory fitness level (CRF): low, moderate, and high. Physical activity (PA) and sitting assessed with the International Physical Activity Questionnaire (IPAQ): A) Sitting time; B) Moderate-to-vigorous PA (MVPA); C) Vigorous PA; and D) Total PA. The high CRF group spent less time in sitting than the other groups (JT= 451.00). Participants in the high CRF group engaged in significantly more MVPA (JT = 972.50), vigorous PA (JT = 1009.00), and total PA (JT = 962.00) than those in the low and moderate CRF groups. However, there were no differences observed between low and moderate CRF in terms of self-reported physical activity metrics. **p ≤ 0.001, *p ≤ 0.01.

High cardiorespiratory fitness and vigorous physical activity relate to select pain sensitivity assessments in healthy adults: A cross-sectional study

Supplemental Materials II

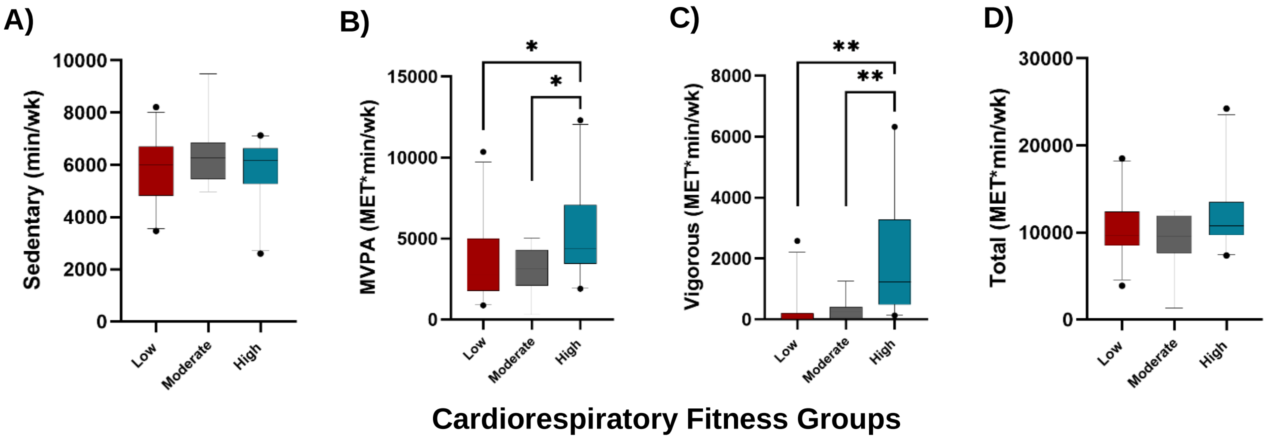

Figure S3. Boxplots (5th, 25th, 50th, 75th, and 95th percentiles shown) of accelerometry-measured activity versus cardiorespiratory fitness (CRF) group (low, moderate, high). Physical activity (PA) assessed with accelerometry: A) Sedentary time; B) Moderate-to-vigorous PA (MVPA); C) Vigorous PA; and D) Total PA. Participants in the high CRF group engaged in significantly more MVPA (JT = 714.00) and vigorous PA (JT = 907.00) than those in the low and moderate CRF groups. However, total PA and the time spent sedentary (JT= 579.00) were not significantly different across CRF categories. Further, no differences in activity were observed between the low and moderate fitness levels. **p ≤ 0.001, *p ≤ 0.01.

High cardiorespiratory fitness and vigorous physical activity relate to select pain sensitivity assessments in healthy adults: A cross-sectional study

Supplemental Materials II

Table S1. Regression results predicting pressure pain threshold (PPT) by activity level or cardiorespiratory fitness group, considering unadjusted and adjusted models.

	**Unadjusted**				**Adjusted**+			
	Model R^2	Model p-value	Unstandardized β	β P-value	Model R^2	Model p-value	Unstandardized β	β P-value
**CRF**	0.21	<0.001	1.33	**<0.001****	0.34	<0.001	1.25	**0.002****
**IPAQ**								
**Sitting** (hr/wk)	-0.01	0.54	1.00	0.54	0.22	<0.001	1.00	0.67
**MVPA** (METs*hr/wk)	0.11	0.004	1.01	**0.004****	0.29	<0.001	1.00	0.02
**Vigorous** (METs*hr/wk)	0.13	0.002	1.01	**0.002****	0.31	<0.001	1.01	**0.01****
**Total** (METs*hr/wk)	0.07	0.02	1.00	0.02	0.27	<0.001	1.00	0.06
**Accelerometer**								
**Sedentary** (hr/wk)	0.02	0.13	1.01	0.13	0.24	<0.001	1.00	0.25
**MVPA** (METs*hr/wk)	-0.01	0.64	1.01	0.64	0.22	0.002	1.00	0.72
**Vigorous** (METs*hr/wk)	0.08	0.02	1.01	**0.02***	0.28	<0.001	1.01	**0.05***
**Total** (METs*hr/wk)	-0.01	0.55	1.00	0.55	0.22	0.002	1.00	0.71

+Adjusted for sex, positive affect (PA) and negative affect (NA);

PPTs were log transformed to meet normality assumptions, and exponential was employed to reverse log-transformed beta; CRF, cardiorespiratory fitness; IPAQ, International Physical Activity Questionnaire; MVPA, moderate-to-vigorous physical activity; *p ≤ 0.05 (for primary predictor, CRF groups and vigorous PA), **p ≤ 0.01 (for all secondary outcomes).

High cardiorespiratory fitness and vigorous physical activity relate to select pain sensitivity assessments in healthy adults: A cross-sectional study

Supplemental Materials II

Table S2. Logistic regression results predicting temporal summation (TS) incidence (yes ≥ 0.5 change in pain) by activity level and cardiorespiratory fitness group, considering unadjusted and adjusted models.

	Unadjusted			Adjusted+		
	**Odds Ratio**	**$CI_{95\%}$**	**P-value**	**Odds Ratio**	**$CI_{95\%}$**	**P-value**
**CRF**	0.62	(0.26, 1.46)	0.28	0.56	(0.20, 1.54)	0.26
**IPAQ**						
**Sitting** (hr/wk)	1.02	(0.98, 1.06)	0.37	1.01	(0.96, 1.05)	0.74
**MVPA** (METs*hr/wk)	0.98	(0.96, 0.99)	0.02	0.98	(0.96, 0.99)	0.02
**Vigorous** (METs*hr/wk)	0.98	(0.96 1.01)	0.13	0.97	(0.95, 1.00)	0.07
**Total** (METs*hr/wk)	0.98	(0.97, 0.99)	0.02	0.98	(0.97, 0.99)	0.03
**Accelerometer**						
**Sedentary** (hr/wk)	0.97	(0.93, 1.02)	0.22	0.97	(0.92, 1.02)	0.19
**MVPA** (METs*hr/wk)	1.01	(0.99, 1.03)	0.49	1.00	(0.98, 1.03)	0.88
**Vigorous** (METs*hr/wk)	0.99	(0.97, 1.02)	0.50	0.98	(0.95, 1.01)	0.24
**Total** (METs*hr/wk)	1.01	(0.99, 1.02)	0.28	1.01	(0.99, 1.03)	0.37

+Adjusted for sex, positive affect (PA) and negative affect (NA);

CRF, cardiorespiratory fitness; IPAQ, International Physical Activity Questionnaire; MVPA, moderate-to-vigorous physical activity; *p ≤ 0.05 (for primary predictor, CRF and vigorous PA), **p ≤ 0.01 (for all secondary outcomes).

High cardiorespiratory fitness and vigorous physical activity relate to select pain sensitivity assessments in healthy adults: A cross-sectional study

Supplemental Materials II

Table S3. Logistic regression results predicting conditioned pain modulation (CPM-PPT) with pressure pain threshold incidence (CPM-PPT yes ≥ 1 SE change in PPT) by activity level and cardiorespiratory fitness group, considering unadjusted and adjusted models.

	**Unadjusted**			**Adjusted⁺**		
	Odds Ratio	$CI_{95\%}$	P-value	Odds Ratio	$CI_{95\%}$	P-value
**CRF**	0.98	(0.56, 1.69)	0.94	1.09	(0.59, 2.03)	0.69
**IPAQ**						
**Sitting** (hr/wk)	1.01	(0.98, 1.03)	0.69	1.01	(0.98, 1.03)	0.66
**MVPA** (METs*hr/wk)	1.01	(0.99, 1.02)	0.44	1.01	(0.99, 1.02)	0.31
**Vigorous** (METs*hr/wk)	1.00	(0.99, 1.02)	0.77	1.01	(0.99, 1.02)	0.60
**Total** (METs*hr/wk)	1.01	(0.99, 1.02)	0.11	1.01	(0.99, 1.02)	0.07
**Accelerometer**						
**Sedentary** (hr/wk)	0.98	(0.95, 1.00)	0.08	0.97	(0.94, 1.00)	0.07
**MVPA** (METs*hr/wk)	1.00	(0.99, 1.02)	0.52	1.01	(0.99, 1.02)	0.25
**Vigorous** (METs*hr/wk)	0.99	(0.98, 1.02)	0.82	1.00	(0.98, 1.02)	0.99
**Total** (METs*hr/wk)	1.01	(0.99, 1.02)	0.14	1.01	(0.99, 1.02)	0.09

⁺Adjusted for sex, positive affect (PA) and negative affect (NA);

CRF, cardiorespiratory fitness; IPAQ, International Physical Activity Questionnaire; MVPA, moderate-to-vigorous physical activity; *p ≤ 0.05 (for primary predictor, CRF and vigorous PA), **p ≤ 0.01 (for all secondary outcomes).

High cardiorespiratory fitness and vigorous physical activity relate to select pain sensitivity assessments in healthy adults: A cross-sectional study

Supplemental Materials II

Table S4. Logistic regression results predicting conditioned pain modulation (CPM-TS) with temporal summation incidence (CPM-TS yes ≥ 0.5 change in pain) by activity level and cardiorespiratory fitness group, considering unadjusted and adjusted models.

	**Unadjusted**			**Adjusted+**		
	Odds Ratio	$CI_{95\%}$	P-value	Odds Ratio	$CI_{95\%}$	P-value
**CRF**	0.78	(0.45, 1.36)	0.39	0.77	(0.42, 1.42)	0.40
**IPAQ**						
**Sitting** (hr/wk)	1.00	(0.98, 1.03)	0.82	1.00	(0.98, 1.03)	0.89
**MVPA** (METs*hr/wk)	0.99	(0.98, 1.01)	0.36	0.99	(0.98, 1.01)	0.39
**Vigorous** (METs*hr/wk)	0.99	(0.98, 1.01)	0.45	0.99	(0.97, 1.01)	0.41
**Total** (METs*hr/wk)	0.99	(0.99, 1.01)	0.66	0.99	(0.99, 1.01)	0.77
**Accelerometer**						
**Sedentary** (hr/wk)	0.98	(0.96, 1.01)	0.29	0.99	(0.96, 1.02)	0.38
**MVPA** (METs*hr/wk)	1.01	(0.99, 1.03)	0.09	1.01	(0.99, 1.03)	0.16
**Vigorous** (METs*hr/wk)	1.00	(0.98, 1.03)	0.69	1.01	(0.98, 1.03)	0.64
**Total** (METs*hr/wk)	1.01	(0.99, 1.02)	0.17	1.01	(0.99, 1.02)	0.25

+Adjusted for sex, positive affect (PA) and negative affect (NA);

CRF, cardiorespiratory fitness; IPAQ, International Physical Activity Questionnaire; MVPA, moderate-to-vigorous physical activity; *p ≤ 0.05 (for primary predictor, CRF and vigorous PA), *p ≤ 0.01 (for all secondary outcomes).

High cardiorespiratory fitness and vigorous physical activity relate to select pain sensitivity assessments in healthy adults: A cross-sectional study

Supplemental Materials II

Table S5. Sensitivity analyses comparing alternative accelerometry outcomes (steps/day or min/day) with pain sensitivity outcomes using Spearman's Correlation Coefficients (rho).

Variable	PPT	TS	CPM-PPT	CPM-TS
Steps	0.10	-0.02	0.01	0.06
**Freedson's VM3**				
Sedentary	0.07	0.00	-0.06	-0.05
Light PA	-0.24	-0.07	-0.13	0.16
Moderate PA	-0.06	0.03	0.18	0.07
Vigorous PA	0.19	-0.09	0.00	-0.12
**Crouter's VM**				
Sedentary	0.07	0.00	-0.06	-0.05
Light PA	-0.25	-0.07	-0.13	0.15
Moderate PA	-0.06	0.03	0.16	0.06
Vigorous PA	0.21	-0.12	0.00	-0.12
**Nonlinear method**				
Sedentary	0.25	-0.27	-0.20	0.16
Light PA	-0.10	0.17	0.23	-0.06
Moderate PA	-0.11	0.19	0.16	-0.17
Vigorous PA	**0.35***	-0.18	-0.02	0.12

PPTs, pressure pain thresholds; TS, temporal summation; CPM-PPT, conditioned pain modulation with pressure pain threshold, CPM-TS, conditioned pain modulation with temporal summation. *$p \leq 0.01$.
